# Parental chest computerized tomography examination before IVF/ICSI has no impact on pregnancy and neonatal outcomes: a cohort study of 2680 fresh transfer cycles

**DOI:** 10.1186/s12884-022-05297-4

**Published:** 2022-12-26

**Authors:** Lan Wang, Linshuang Li, Yiqing Zhao, Bei Xu, Jing Yue, Hanwang Zhang, Lei Jin

**Affiliations:** Reproductive Medicine Center, Tongji Hospital, Tongji Medical College of Huazhong University of Science and Technology, Wuhan, 430030 China

**Keywords:** Chest computerized tomography, Radiology, IVF/ICSI, Miscarriage, Pregnancy outcome, Neonatal outcome

## Abstract

**Background:**

Some concern has been expressed regarding the negative effects of low-level ionizing radiation exposure in the context of radiological evaluation prior to IVF/ICSI treatment, but the available evidence is limited and conflicting. The aim of this study is to evaluate pregnancy and neonatal outcomes of couples who did chest computed tomography (CT) prior to IVF/ICSI.

**Methods:**

This was a retrospective cohort study of 2680 IVF/ICSI fresh embryo transfer cycles conducted from January 2019 – August 2020. Fertility outcomes were compared between couples that had or had not undergone CT examination within 3 months prior to the date of oocyte retrieval and sperm collection. Miscarriage was the primary study outcome, while secondary outcomes included the number of oocytes collected, oocyte maturation, normal fertilization, number of good quality cleavage stage embryos, blastocyst formation, implantation, clinical pregnancy, ectopic pregnancy, live birth, multiple birth, Cesarean section rates, gestational weeks, maternal obstetric complications, birth weight, newborn sex ratio, and birth defect incidence. Propensity score matching was used to control for potential confounding variables.

**Results:**

Of the 2680 cycles included in this study, couples underwent CT examination in 731 cycles. After 1:1 propensity score matching, 670 cycles were included in each group. When comparing demographic and fertility-related variables between groups that had and had not undergone CT examination after propensity score matching, we detected no significant differences in miscarriage rates (16.99% vs. 15.77%, OR = 1.10, 95CI% = 0.74 to 1.68). Similarly, both groups exhibited comparable oocyte and embryonic development, implantation rates (41.99% vs. 40.42%, OR = 1.07, 95%CI = 0.87 to 1.31), clinical pregnancy rates (45.67% vs. 44.48%, OR = 1.05, 95%CI = 0.85 to 1.30), ectopic pregnancy rates (2.94% vs. 1.68%, OR = 1.78, 95%CI = 0.59 to 5.36), live birth rates (36.57% vs. 35.67%, OR = 1.04, 95%CI = 0.83 to 1.30), multiple birth rates, Cesarean section rates, gestational weeks, maternal obstetric complication rates, and neonatal outcomes.

**Conclusions:**

Chest CT examination before IVF/ICSI has no impact on pregnancy and neonatal outcomes associated with fresh embryo transfer.

**Trial registration:**

Not applicable.

## Background

Chest computed tomography (CT) examinations are among the most common forms of hospital examinations, particularly in the context of the COVID-19 pandemic [1–3], with chest CT scans and nucleic acid testing being routinely used to screen for COVID-19 pneumonia prior to other medical procedures as a means of minimizing the risk of hospital infection [4]. Such scans may also be conducted prior to oocyte retrieval and sperm collection among women undergoing in vitro fertilization (IVF) or intracytoplasmic sperm injection (ICSI). Exposure to ionizing radiation during the preconception period is an important consideration for these women, and whether preconception maternal and/or paternal chest CT scans can adversely impact embryonic development, clinical pregnancy outcomes, and neonatal outcomes for couples undergoing IVF/ICSI cycle remains controversial. Pelvic radiotherapy is already known to be potentially damaging to gamete competency and reproductive outcomes [5]. It is thus unsurprising that concern exists regarding the potential adverse effects of chest CT scans on gamete development and fertility outcomes. However, overestimating such risk has the potential to deprive couples of otherwise beneficial imaging analyses, and may lead certain couples to delay fertility treatment following ionizing radiation exposure. To date there have been few studies on this topic, as couples generally avoid undergoing chest CT scanning prior to IVF/ICSI other than in emergency contexts, underscoring a key knowledge gap in the current literature.

In vitro*,* both spermatogonia and oogonia are highly sensitive to ionizing radiation exposure [6, 7], and there is strong evidence that environmental, physical, and chemical stressors including ionizing radiation can adversely impact male fertility [8]. Early epidemiological evidence suggested that as little as 0.11 Gy was sufficient to significantly suppress human spermatogenesis [9], with in vitro evidence that a dose as low as 10 mGy can arrest spermatogenesis [10]. Higher rates of mosaicism and hypoploidy were observed in murine embryos derived from spermatozoa exposed to ionizing radiation as compared to control spermatozoa when studying chromosomal abnormalities in early cleavage stage embryos [11]. Despite these findings, however, epidemiological evidence also suggests that males are more resistant than females with respect to radiological exposure. An occupational cohort study of low-level ionizing radiation exposure detected no association between such exposure prior to conception and the odds of adverse reproductive outcomes in men, whereas in women such exposure was related to higher rates of miscarriage and stillbirth [12].

Animal examinations showed that radiosensitivity of the oocytes varies widely according to different stage of follicles [13]. Preovulatory oocytes are also known to be more radiosensitive as compared to primordial oocytes [13–15], and oocyte damage in response to radiation is both dose- and age-dependent [15]. Parental irradiation can introduce multiple mutations and small deletions within oocytes and spermatagonia in mammals [7]. However, it is difficult to extrapolate the results of these preclinical and in vitro studies owing to the high doses of acute radion used therein that do not reflect the lower doses utilized in chest CT scans and other human imaging applications. Indeed, chest CT scans incur a radiation dose of approximately 8.0 mGy, which is well below the damaging doses in these studies [13]. The radiation necessary to kill 50% of oocytes in the human ovary (LD_50_) is 2 Gy [15, 16]. As such, it is theoretically safe to perform chest CT scans prior to oocyte retrieval and sperm collection during IVF/ICSI treatment. However, couples and their clinicians nonetheless tend to express concern regarding potential adverse outcomes associated with chest CT scanning immediately prior to fertility treatment.

At present, there is a lack of standardized CT examination guidelines for patients before IVF/ICSI procedures. This study was developed to provide clinical evidence regarding the impact of chest CT scans prior to IVF/ICSI on embryonic development, pregnancy outcomes, and neonatal complications. To that end, we conducted a retrospective analysis of 2680 fresh embryo transfer cycles in which both the male and female partners had or had not undergone chest CT examinations within 3 months prior to IVF/ICSI treatment in our reproductive medicine center with the goal of establishing the impact of chest CT scans on embryonic, pregnancy, and neonatal outcomes following IVF/ICSI.

## Methods

This was a retrospective cohort study of patients undergoing IVF/ICSI from January 2019 – August 2020. This study was conducted in a manner consistent with the standards for reporting observational studies (STROBE) [17].

### Study population

**Fig. 1:**
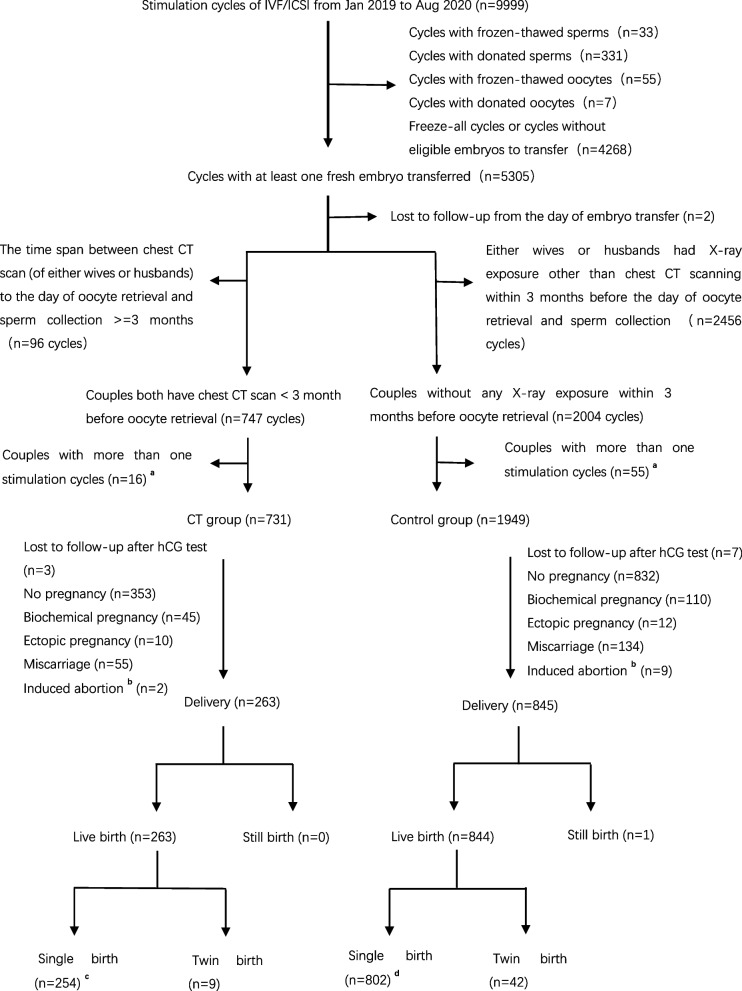
This is the flow chart of this study. Steps with superscript (labeled as a, b, c, d) were explained as following. **a** Only the first cycle of couples with more than one stimulation cycles was included in the final analysis. **b** The reason of induced abortion was fetal abnormality diagnosed by amniocentesis (8 cases) and unknown (3 cases). **c** One woman had twin pregnancy, but one of the fetus was missed before 12 gestational weeks. **d** Two women had twin pregnancy and both had elective embryo reduction in the first term of pregnancy

All IVF/ICSI patients that underwent fresh embryo transfer cycles in the reproductive medical center of Tongji Hospital, Tongji Medical College of Huazhong University of Science and Technology (Wuhan, China) from January 2019 – August 2020 were initially included in this study (Fig. 1). Couples were included in the CT group if both had undergone chest CT scanning within the three-month period prior to egg retrieval, or to the control group if both had not undergone such evaluation. Patients were excluded from the study if they had undergone chest CT scans other than during this period or if they had undergone other forms of radiological examination such as X-rays. CT scanning indications included COVID-19 screening prior to oocyte retrieval and sperm collection. No included patients were positive for COVID-19. Only cycles in which a minimum of one fresh embryo was transferred were included in these analyses. Only the first stimulation cycle from each couple was analyzed. Patients were excluded if their data were incomplete, and patient follow-up was conducted until delivery via phone call.

### Treatment characteristics

Ovarian stimulation protocols for patients in the present study included the short GnRH agonist protocol, GnRH antagonist protocol, long GnRH agonist protocol, mild stimulation or natural cycle protocols as discussed previously [18–20]. Patients were administered a daily 150–300 IU injection of recombinant follicle-stimulating hormone (FSH), highly purified human menopausal gonadotropin (hMG), or recombinant FSH and hMG. Ovarian stimulation protocols were selected based upon maternal age, maternal BMI, ovarian reserve, history of treatment, and physician experience. Individual ovarian responses were assessed to adjust gonadotropin dosing.

Either hCG or GnRH agonist were used to trigger oocyte maturation when a minimum of 2 leading follicles at least 18 mm in diameter were evident, with oocyte retrieval and sperm collection then being performed 34–36 h later. After collection, oocytes were subjected to IVF/ICSI insemination, with zygotes being cultured until Day 3 or the blastocyst stage. Embryo transfer was then conducted with ultrasonographic guidance. Fresh transfer cycles were canceled when patients were considered to be at risk of ovarian hyperstimulation syndrome, exhibited premature increases in progesterone levels on the day of hCG trigger (over 1.5 ng/mL), had endometrial polyps or intrauterine fluid, or elected to undergo preimplantation genetic testing. ASRM guidelines were used to select the number of embryos for transfer [21]. Intravaginal progesterone and oral dydrogesterone were administered to provide luteal phase support from the day of oocyte retrieval until week 6 following embryo transfer, with pregnancies being monitored until delivery based on established protocols [18, 22].

### Chest CT scanning

Chest CT scans were performed with patients in the supine position with their arms raised using one of the following instruments: uCT 780, General Electric Company, USA; Somatom Force, Siemens Healthcare, Germany. Primary scanning parameters were as follows: tube voltage = 120 kVp, automatic tube current modulation, pitch = 0.99–1.22 mm, matrix = 512 × 512, slice thickness = 10 mm, field of view = 350 mm × 350 mm. Intravenous iodinated contrast medium was not used during scanning for any patients included in this study.

### Data input

Demographic data including age, body mass index (BMI), basal FSH, antra follicle count (AFC), anti-Mullerian hormone (AMH), primary/secondary infertility, duration of infertility, and cause of infertility were analyzed for this study, as were treatment data including stimulation scheme, mean dose of Gonadotropin per day, endometrial thickness on the day of hCG injection, progesterone level on the day of hCG injection, manner of insemination (IVF or ICSI), and number of embryos transferred.

### Outcome measurements

Miscarriage was the primary outcome of this study and was defined as a pregnancy loss prior to 22 weeks of gestation, with miscarriage rates being calculated as a fraction of the overall population of pregnant women [23]. Secondary outcomes included the number of oocytes retrieved, number of matured (MII phase) oocytes, normal fertilization, number of good quality cleavage stage embryos, blastocyst formation, implantation rates, clinical pregnancy rates, ectopic pregnancy rates, live birth rates, multiple birth rates, Cesarean section rates, gestational weeks, maternal obstetric complication rates, birth weight, newborn gender ratios, and neonatal birth defect rates. Implantation rates were confirmed when the gestational sac was visible upon ultrasonographic assessment, and were calculated based on the number of observed gestational sacs divided by the number of transferred embryos [23]. Clinical pregnancy was confirmed based upon the detection of fetal cardiac activity as detected via ultrasonography 4 to 6 weeks following transplantation [24], with these rates being calculated on a per transfer cycle basis. Ectopic pregnancies were defined as pregnancies wherein implantation occurred outside of the uterine cavity as detected via ultrasonography, with these rates being calculated as a fraction of the overall population of pregnant women [23]. Live births were defined as a live birth of a baby after 22 weeks gestational age [23], with these rates being calculated as the number of live births per transfer cycle. Multiple birth was defined as the delivery of more than one fetus after 22 weeks gestational age [23], with this rate being calculated on a per live birth basis.

### Statistical analysis

Our sample size was calculated based on the ability to detect a 5% difference in miscarriage rates from the 13% baseline rate at a power level of 80% and an alpha = 0.05, yielding a calculated sample size of 644 per group. Continuous data were given as means with standard deviations or medians with the interquartile range, while categorical data were given as percentages. Data were compared via Mann-Whitney U tests, Pearson chi-squared tests, and Wilcoxon tests as appropriate. Missing values are listed in tables and were replaced with the corresponding mean value.

Propensity score matching (PSM) was used to control for potential confounding factors. Briefly, 1:1 PSM without replacement was performed for baseline patient characteristics including age, BMI, basal FSH, AFC, anti-Mullerian hormone, primary/secondary infertility, duration of infertility, cause of infertility, stimulation scheme, mean dose of Gonatropin per day, endometrial thickness on the day of hCG injection, progesterone level on the day of hCG injection, manner of insemination (IVF or ICSI), and the number of embryos transferred. Data were then compared between groups following PSM, with odds ratios (ORs) and 95% confidence intervals (CIs) being calculated. SPSS 26.0 (SPSS, IBM, USA) was used for all statistical testing, with a two-tailed *P* < 0.05 as the significance threshold.

## Results

In total, 9999 stimulation cycles were performed at our center from January 2019 – August 2020, of which 5305 included the transfer of at least one fresh embryo. Of these 5305 transfer cycles, 747 cycles included couples that had both undergone chest CT scans within 3 months prior to the day of oocyte retrieval and sperm collection, and 2004 cycles included couples that had not undergone any diagnostic radiological assessment during this same time period. Only the first cycle was included in this analysis for couples that underwent more than one stimulation cycle (Fig. 1). In total, 731 and 1949 cycles were included in the CT and control groups in these analyses. All couples were followed until delivery, with neonatal birth defects also being recorded.

Demographic data pertaining to the couples eligible for inclusion in these analyses are compiled in Table 1. Overall, women in the CT group were older (31.54 ± 4.18 vs. 30.99 ± 4.01 years old, *P* = 0.003), had a lower BMI (21.91 ± 3.05 vs. 22.13 ± 3.05, *P* = 0.046), had fewer antral follicles (11.66 ± 6.45 vs. 13.62 ± 6.54, *P* < 0.001), lower serum AMH levels (3.13 ± 2.66 vs. 4.90 ± 3.88 ng/ml, *P* < 0.001), and a shorter infertility duration (2.91 ± 2.21 vs. 3.37 ± 2.46 years, *P* < 0.001) relative to those of patients in the control group. Moreover, women in the CT group exhibited fewer ovulatory disorders (9.03% vs. 17.5%, *P* < 0.001), fewer uterus-related infertility factors (4.79% vs. 9.13%, *P* < 0.001) and greater decreases in ovarian reserve (18.19% vs. 13.03%, *P* = 0.001) relative to those of control women. There were also differences in primary ovarian stimulation scheme and daily gonadotropin dose between these groups, with women in the CT group having a thinner endometrium (11.06 ± 2.21 vs. 11.72 ± 2.51, *P* < 0.001) and higher serum progesterone levels (0.73 ± 0.36 vs. 0.69 ± 0.36, *P* = 0.009) on the day of hCG administration as compared to controls. The proportion of women undergoing single embryo transplantation was also higher in the CT group (87.41% vs. 82.14%, *P* = 0.001). Propensity score matching (PSM; 1:1) was then conducted to minimize the potentially confounding effects of the above baseline characteristics. Following PSM, 1340 cycles (670 cycles per group) were retained for subsequent analysis (Table 1).

**Table 1:** Baseline characteristic of fresh cycles before and after matching. Values are presented as mean + standard deviation or fraction (%)

Demographic parameters (missing data %)	Before propensity Score Matching			After propensity Score Matching		
	CT group	Control group	*P*-value	CT group	Control group	*P*-value
Number of cycles	731	1949		670	670	
Age	31.54 ± 4.18	30.99 ± 4.01	0.003	31.51 ± 4.22	31.66 ± 4.27	0.593
Body mass index	21.91 ± 3.05	22.13 ± 3.05	0.046	21.91 ± 3.09	21.79 ± 2.97	0.645
Basal FSH (0.04%)	7.78 ± 2.63	7.57 ± 2.44	0.234	7.78 ± 2.68	7.90 ± 2.77	0.275
Antra follicle count (0.15%)	11.66 ± 6.45	13.62 ± 6.54	< 0.001	11.66 ± 6.36	11.41 ± 5.88	0.454
Anti-Mullerian hormone (0.78%)	3.13 ± 2.66	4.90 ± 3.88	< 0.001	3.19 ± 2.71	3.19 ± 2.34	0.780
Primary/secondary infertility	731	1949	0.295	670	638	0.730
primary, n%	494 (67.58)	1358 (69.68)		447 (66.57)	440 (65.67)	
secondary, n%	237 (32.42)	591 (30.32)		223 (33.43)	230 (34.33)	
Duration of infertility	2.91 ± 2.21	3.37 ± 2.46	< 0.001	2.99 ± 2.25	3.12 ± 2.20	0.157
Cause of infertility						
ovulation disorder, n%	66 (9.03)	341 (17.50)	< 0.001	63 (7.61)	58 (8.66)	0.699
Pelvic factor, n%	339 (46.37)	920 (47.20)	0.702	307 (48.10)	305 (45.52)	0.955
Uterine factor, n%	35 (4.79)	178 (9.13)	< 0.001	35 (5.37)	45 (6.72)	0.289
Male factor, n%	194 (26.54)	589 (30.22)	0.062	185 (28.21)	175 (26.12)	0.584
Endometriosis, n%	64 (8.76)	137 (7.03)	0.131	58 (8.66)	60 (8.96)	0.923
Decreased ovarian reserve, n%	133 (18.19)	254 (13.03)	0.001	120 (17.91)	132 (19.70)	0.432
Unexplained, n%	78 (10.67)	233 (11.95)	0.355	76 (11.34)	70 (10.45)	0.653
Stimulation scheme	731	1949	< 0.001	670	670	0.799
short GnRH agonist protocol, n%	71 (9.71)	135 (6.93)		71 (10.60)	74 (11.04)	
GnRH antagonist protocol, n%	483 (66.07)	669 (34.33)		422 (62.99)	427 (63.73)	
long GnRH agonist protocol, n%	174 (23.81)	1130 (57.98)		174(25.96)	167 (24.93)	
mild stimulation or natural cycle protocol, n%	3 (0.41)	15 (0.76)		3 (0.45)	2 (0.30)	
Mean dose of Gonadotropin per day, IU	260.63 ± 65.08	245.35 ± 75.05	< 0.001	260.60 ± 66.20	263.72 ± 73.88	0.458
Endometrial thickness on the day of hCG injection, mm	11.06 ± 2.21	11.72 ± 2.51	< 0.001	11.13 ± 2.23	11.22 ± 2.39	0.724
Progesterone level on the day of hCG injection, ng/ml (0.15%)	0.73 ± 0.36	0.69 ± 0.36	0.009	0.72 ± 0.37	0.72 ± 0.39	0.716
Manner of insemination (IVF/ICSI)	731	1949	0.017	670	670	0.361
IVF, n%	471 (64,43)	1157 (59.36)		419 (62.54)	436 (65.07)	
ICSI, n%	260 (35.57)	792 (40.64)		251 (37.46)	234 (34.93)	
Number of embryos transferred	731	1949	0.001	638	638	0.692
1, n%	639 (87.41)	1601 (82.14)		578 (86.27)	573 (85.52)	
2, n%	92 (12.59)	348 (17.86)		92 (13.73)	97 (14.48)	

Data pertaining to embryo development are shown in Table 2. Following PSM, women in the CT and control groups exhibited similar numbers of retrieved oocytes (10.60 ± 5.08 vs. 10.47 ± 5.32, *P* = 0.418), matured oocytes (MII oocytes, 8.91 ± 4.41 vs. 8.78 ± 4.76, *P* = 0.456), normal fertilized embryos with two pronuclei (6.31 ± 3.56 vs. 6.13 ± 3.85, *P* = 0.175), good quality cleavage stage embryos (3.32 ± 2.54 vs.3.22 ± 2.72, *P* = 0.244), and blastocyst embryos (4.03 ± 3.00 vs. 3.79 ± 3.27, *P* = 0.055) (Table 2).

**Table 2:** Embryo development of fresh cycles before and after matching

	Before propensity Score Matching			After propensity Score Matching		
	CT group	Control group	*P*-value	CT group	Control group	*P*-value
Number of cycles	731	1949		670	670	
Number of Oocytes Retrieved	10.60 ± 5.04	11.77 ± 5.40	0.000	10.60 ± 5.08	10.47 ± 5.32	0.418
MII oocytes ^a^	8.93 ± 4.38	9.75 ± 4.72	0.000	8.91 ± 4.41	8.78 ± 4.76	0.456
2PN embryos ^b^	6.34 ± 3.57	6.90 ± 3.90	0.002	6.31 ± 3.56	6.13 ± 3.85	0.175
Good quality cleavage stage embryos ^c^	3.35 ± 2.53	3.61 ± 2.78	0.066	3.32 ± 2.54	3.22 ± 2.72	0.244
Embryos continued cultured from Day three	5.78 ± 3.423	6.41 ± 3.79	0.001	5.76 ± 3.45	5.80 ± 3.80	0.236
Blastocyst	4.04 ± 3.00	4.27 ± 3.30	0.290	4.03 ± 3.00	3.79 ± 3.27	0.055

^a^ MII means metaphase II

^b^ 2PN means 2 pronuclei

^c^ Good quality cleavage stage embryos were defined as embryos which have 2 pronuclei on day 0, 4–5 cells on day 2, 7–10 cells with even blastomeres and less than 10% fragment on day 3

Pregnancy outcomes for both patient groups were also collected (Table 3). After PSM, there were no significant differences in miscarriage rates between groups (16.99% vs. 15.77%, OR = 1.10, 95CI% = 0.74 to 1.68). Similarly, implantation rates (41.99% vs. 40.42%, OR = 1.07, 95%CI = 0.87 to 1.31), clinical pregnancy rates (45.67% vs. 44.48%, OR = 1.05, 95%CI = 0.85 to 1.30), ectopic pregnancy rates (2.94% vs. 1.68%, OR = 1.78, 95%CI = 0.59 to 5.36), live birth rates (36.57% vs. 35.67%, OR = 1.04, 95%CI = 0.83 to 1.30), multiple birth rates (3.67% vs 4.18%, OR = 0.87, 95%CI = 0.35 to 2.19), Cesarean section rates (73.06% vs. 69.46%, OR = 1.19, 95%CI = 0.80 to 1.77), gestational weeks (37.86 ± 2.24 vs. 38.49 ± 1.46, *P* = 0.173), and maternal obstetric complication rates (9.80% vs. 6.69%, *P* = 0.215) were comparable in both groups. With respect to newborns, there were no significant differences in birth weight (3276.02 ± 1752.75 vs. 3241.89 ± 429.292 g, *P* = 0.589), sex ratios (144 to 110 vs. 122 to 127, *P* = 0.084), or birth defect rates (0.79% vs. 0.80%, *P* = 0.984) when comparing these groups (Table 4).

**Table 3:** Clinical outcomes of fresh cycles before and after matching

	Before propensity Score Matching				After propensity Score Matching			
	CT group	Control group	OR (95%CI)	*P*-value	CT group	Control group	OR (95%CI)	*P*-value
Number of cycles	731	1949			670	670		
Implantation rate, n%	41.68 (343/823)	46.19 (1061/2297)	0.832 (0.71,0.98)	0.026	41.99 (320/762)	40.42 (310/767)	1.07 (0.87/1.31)	0.531
Clinical pregnancy rate, n%	44.87 (328/731)	51.67 (1007/1949)	0.76 (0.64,0.90)	0.002	45.67 (306/670)	44.48 (298/670)	1.05 (0.85,1.30)	0.661
Miscarriage rate, n%	16.77 (55/328)	13.31 (134/1007)	1.31 (0.932,1.848)	0.118	16.99 (52/306)	15.77 (47/298)	1.10 (0.74,1.68)	0.685
Ectopic pregnancy rate, n%	3,05 (10/328)	1.19 (12/1007)	2.61 (1.12,6.09)	0.022	2.94 (9/306)	1.68 (5/298)	1.78 (0.59,5.36)	0.302
Live birth rate, n%	35.98 (263/731)	43.30 (844/1949)	0.74 (0.62,0.88)	0.001	36.57 (245/670)	35.67 (239/670)	1.04 (0.83,1.30)	0.733
Multiple birth rate, n%	3.42 (9/263)	4.98 (42/844)	0.68 (0.33,1.41)	0.294	3.67 (9/245)	4.18 (10/239)	0.87 (0.35,2.19)	0.772
Cesarean section rate, n%	73.00 (192/263)	73.22 (618/844)	0.99 (0.72,1.35)	0.944	73.06 (179/245)	69.46 (166/239)	1.19 (0.80,1,77)	0.381

**Table 4:** Perinatal outcomes before and after matching

	Before propensity Score Matching			After propensity Score Matching		
	CT group	Control group	*P*-value	CT group	Control group	*P*-value
Number of cycles	731	1949		670	670	
Still birth, n‰	0 (0)	1 (1.13)	0.579	0 (0)	0 (0)	
Gestational age (weeks)	37.87 ± 2.23	38.34 ± 1.74	0.001	37.86 ± 2.24	38.49 ± 1.46	0.173
Gender ^a^			0.109			0.084
male, n(%)	154 (56.62)	452 (51.02)		144 (56.69)	122 (49.00)	
female, n(%)	118 (43.38)	433 (48.87)		110 (43.31)	127 (51.00)	
Weight (g) ^b^	3273.24 ± 1701.92	3229.60 ± 503.22	0.267	3276.02 ± 1752.75	3241.89 ± 429.292	0.589
Maternal obstetric complications ^c^, n(%)	26 (9.89)	72 (8.52)	0.496	24 (9.80)	16 (6.69)	0.215
Neonatal birth defect ^d^, n(%)	2 (0.74)	7 (0.79)	0.927	2 (0.79)	2 (0.80)	0.984

^a^ The gender datum of one baby in the control group is missing

^b^ The birth weight datum of one baby in the control group is missing

^c^ The main maternal obstetric complication included placenta previa, premature rupture of amniotic fluid, gestational hypertension, gestational diabetes, hyperthyroidism, hypothyroidism, and so on

^d^ The neonatal birth defect included pyloric stenosis (1 case), cardiac malformation (4 cases), cheiloschisis (1 case), auricle deformity (2 cases), and hemangioma of the finger (1 case)

## Discussion

Herein, we determined that parental chest CT examination prior to IVF/ICSI had no adverse impact on embryo development, with the numbers of oocytes retrieved, matured oocytes, normal fertilization, good quality cleavage embryos, and blastocyst formation all being comparable between couples that had undergone chest CT scans and control couples. Moreover, both of these study groups exhibited similar pregnancy outcomes including miscarriage, implantation, clinical pregnancy, ectopic pregnancy, live birth, multiple birth, Cesarean section, and maternal obstetric complication rates. With respect to neonatal outcomes, no differences in gestational weeks, birth weights, sex ratios, or birth defects were observed when comparing newborns in these two groups.

CT imaging is integral to the diagnosis of a variety of chronic and acute diseases and health conditions. In couples seeking to become pregnant, radiation exposure during the preconception period is often a source of anxiety [25, 26], particularly for couples awaiting IVF/ICSI treatment. However, prior studies have not clarified the effects of CT scanning on embryonic development, pregnancy outcomes, and neonatal complications associated with IVF/ICSI. Epidemiological studies have primarily focused on pregnancy outcomes associated with diagnostic radiology during pregnancy or on the overall effects of radiation on general populations following nuclear accidents. In an early case-control study, intrauterine exposure to one pelvic CT scan during pregnancy was shown to increase the risk of cancer in exposed offspring later in life [27]. This suggests that fetuses exhibit a higher degree of radiosensitivity than do adults [28]. However, chest CT scans are distinct from abdominal and pelvic CT scans, and cohort studies of pregnant populations have not reported any adverse effects of chest CT scans on pregnancy outcomes [25, 29, 30]. Epidemiological data pertaining to high-profile radiation exposure such as that associated with atomic bombings or the Chernobyl reactor accident, while emotionally salient, also suggest that birth defects only arise following high-level radiation exposure (> 100 mGy) which does not occur in the context of a single CT examination [26].

Adverse outcome risk associated with radiation exposure is dose-dependent [31], and no current evidence suggests that human exposure to diagnostic radiation doses (< 0.5 Gy) before or during pregnancy can increase the risk of embryonic loss, growth retardation, or congenital malformation [25]. Prior research suggests that estimated radiation doses to the conceptus from chest CT are < 0.1 mGy [30], suggesting that such CT scans should theoretically have negligible reproductive influence. However, no guidelines exist regarding what levels of radiation exposure are safe prior to or during pregnancy given that certain radiation-related effects are stochastic in nature. Herein, we found that embryonic development dynamics were normal following parental chest CT exposure within 3 months before the day of oocyte retrieval and sperm collection as compared to that observed for control individuals that did not undergo CT examination. CT scans had no impact on pregnancy outcomes or neonatal complications in this study population. Rates of miscarriage were similar in both study groups. In one prior epidemiological analysis of males exposed to high levels of natural background radiation, tandem duplication and copy number polymorphisms of the SRY gene were observed [32]. However, we did not detect any differences in newborn sex ratios when comparing the CT and control groups in this study.

This study is subject to a number of limitations. For one, this was a retrospective analysis, and we did not collect data pertaining to oocyte and sperm quality prior to and after chest CT scanning. Moreover, we did not specifically measure the radiation dose to which oocytes and sperm were exposed prior to IVF/ICSI treatment. Furthermore, the effects of ionising radiation on gametes may not be limited to the 3 months before treatment. In addition, this study was conducted during the COVID-19 pandemic, which may have introduced other confounding variables into the study such as changes in the psychological status of patients and medical staff that may have impacted IVF/ICSI outcomes. We also only followed patients until delivery, and certain congenital malformations or epigenetic disorders may only manifest months or years after birth. Further research with a longer follow-up period is thus required to more fully gauge the risks associated with chest CT scanning in the context of fertility treatment. In the current study, we failed to analyze the individual impact of maternal or paternal CT exposure separately, because both female and male partner needed to pass the chest CT examination and nucleic acid test prior to IVF/ICSI in order to avoid hospital infection during COVID-19 pandemic. The external validity of this study may also be limited, given that in many countries CT scans would not be used as widely as is described here during and after the pandemic.

In addition to being used to screen for COVID-19 pneumonia, chest CT scanning is routinely conducted to detect and evaluate many other clinically relevant conditions such as tuberculosis, lung cancer, and trauma. Our results suggest that there is no need to postpone oocyte retrieval protocols for women that had undergone a single chest CT examination. As clinical data and guidance for women seeking to undergo IVF/ICSI following ionizing radiation exposure are limited, these findings can aid clinicians in patient counseling and management efforts. However, caution should be taken when interpreting the results of this study, and the potential negative effects of chest CT scanning before IVF/ICSI should always be balanced against the perceived benefits of such imaging. Further prospective research on this topic will be necessary to fully clarify the long-term impact of radiation exposure on newborn well-being.

## Conclusion

Chest CT exposure of both couples 3 months prior to the day of oocyte retrieval and sperm collection has no adverse effect on the pregnancy and neonatal outcome of fresh embryo transfer cycle in IVF/ICSI. It is not necessary for couples to postpone ovarian stimulation and IVF/ICSI after chest CT examination.

## Data Availability

The datasets generated and analysed during the current study are not publicly available due to individual privacy but are available from the corresponding author on reasonable request.

## Abbreviation

(CT): Chest computed tomography
(IVF): In vitro fertilization
(ICSI): Intracytoplasmic sperm injection
(FSH): Follicle-stimulating hormone
(hMG): Human menopausal gonadotropin
(BMI): Body mass index
(AFC): Antra follicle count
(AMH): Anti-Mullerian hormone
(PSM): Propensity score matching
(ORs): Odds ratios
(CIs): Confidence intervals
